# Neurogenetic and Epigenetic Aspects of Cannabinoids

**DOI:** 10.3390/epigenomes6030027

**Published:** 2022-08-26

**Authors:** Catherine A. Dennen, Kenneth Blum, Abdalla Bowirrat, Jag Khalsa, Panayotis K. Thanos, David Baron, Rajendra D. Badgaiyan, Ashim Gupta, Eric R. Braverman, Mark S. Gold

**Affiliations:** 1 Department of Family Medicine, Jefferson Health Northeast, Philadelphia, PA 19114, USA; 2 Division of Nutrigenomics, The Kenneth Blum Behavioral Neurogenetic Institute, Austin, TX 78701, USA; 3 Division of Addiction Research & Education, Center for Psychiatry, Medicine & Primary Care (Office of the Provost), Graduate College, Western University of Health Sciences, Pomona, CA 91766, USA; 4 Institute of Psychology, ELTE Eötvös Loránd University, 1075 Budapest, Hungary; 5 Department of Psychiatry, University of Vermont, Burlington, VT 05405, USA; 6 Department of Psychiatry, Wright University Boonshoft School of Medicine, Dayton, OH 45324, USA; 7 Department of Molecular Biology, Adelson School of Medicine, Ariel University, Ariel 40700, Israel; 8 Department of Microbiology, Immunology and Tropical Medicine, School of Medicine and Health Sciences, The George Washington University, Washington, DC 20037, USA; 9 Medical Consequences of Drug Abuse and Infections Branch, National Institute on Drug Abuse, National Institutes of Health, Bethesda, MD 20852, USA; 10 Behavioral Neuropharmacology and Neuroimaging Laboratory on Addictions, Clinical Research Institute on Addictions, Department of Pharmacology and Toxicology, Jacobs School of Medicine and Biosciences, State University of New York at Buffalo, Buffalo, NY 14260, USA; 11 Department of Psychiatry, Icahn School of Medicine Mt Sinai, New York, NY 10029, USA; 12 Department of Psychiatry, South Texas Veteran Health Care System, Audie L. Murphy Memorial VA Hospital, San Antonio, TX 78229, USA; 13 Long School of Medicine, University of Texas Medical Center, San Antonio, TX 78229, USA; 14 Future Biologics, Lawrenceville, GA 30043, USA; 15 Department of Psychiatry, Washington University School of Medicine, St. Louis, MO 63110, USA

**Keywords:** cannabis, cannabinoids, Cannabis Use Disorder (CUD), epigenetics, Reward Deficiency Syndrome (RDS)

## Abstract

Cannabis is one of the most commonly used and abused illicit drugs in the world today. The United States (US) currently has the highest annual prevalence rate of cannabis consumption in the world, 17.9% in individuals aged 12 or older, and it is on the rise. With increasing cannabis use comes the potential for an increase in abuse, and according to the Substance Abuse and Mental Health Services Administration (SAMHSA), approximately 5.1% of Americans had Cannabis Use Disorder (CUD) in 2020. Research has shown that genetics and epigenetics play a significant role in cannabis use and CUD. In fact, approximately 50–70% of liability to CUD and 40–48% of cannabis use initiation have been found to be the result of genetic factors. Cannabis usage and CUD have also been linked to an increased risk of psychiatric disorders and Reward Deficiency Syndrome (RDS) subsets like schizophrenia, depression, anxiety, and substance use disorder. Comprehension of the genetic and epigenetic aspects of cannabinoids is necessary for future research, treatment plans, and the production of pure cannabinoid compounds, which will be essential for FDA approval. In conclusion, having a better understanding of the epigenetic and genetic underpinnings of cannabis use, CUD, and the endocannabinoid system as a whole will aid in the development of effective FDA-approved treatment therapies and the advancement of personalized medicine.

## 1. Introduction

Cannabis is one of the most commonly used and abused illicit drugs in the world today. According to estimates from the World Health Organization (WHO) and the United Nations (UN), approximately 150–200 million people worldwide consume cannabis annually, and young adults (aged 18–25) are the most common users [1,2]. The United States (US) currently has the highest annual prevalence rate of cannabis consumption in the world, and it is on the rise [1,2,3]. According to the Substance Abuse and Mental Health Services Administration (SAMHSA), in the US in 2020, annual cannabis consumption increased from 17.5% (2019) to 17.9% in individuals aged 12 or older (~49.6 million people) [3]. Young adults (aged 18–25) had the highest prevalence rate of cannabis use at 34.5% (11.6 million people), followed by adults (aged 26 or older) at 16.3% (35.5 million people), and then adolescents (aged 12–17) at 10.1% (2.5 million people) [3].

With increasing cannabis use comes the potential for an increase in abuse. Cannabis Use Disorder (CUD) is diagnosed in the fifth edition of the Diagnostic and Statistical Manual of Mental Disorders (DSM–5) if at least two of eleven official criteria are met within a 12-month period, which includes pathological patterns such as impaired control, physiological adaptation, social impairment, or risky behavior [4]. Furthermore, CUD can be classified by severity. For example, mild is defined as 2–3 symptoms, moderate is 4–5 symptoms, and severe is 6 or more symptoms. According to SAMHSA, approximately 5.1% (14.2 million) of Americans had CUD in 2020 [3]. Young adults (aged 18–25) had the highest prevalence rate of CUD at 13.5% (4.5 million people), followed by adolescents (aged 12–17) at 4.1% (1.0 million people), and adults (aged 26 or older) at 4% (8.7 million people) [3].

The expanding consumption and legalization of marijuana has led to a renewed interest in the therapeutic effects of cannabis. Cannabis has a number of potential therapeutic benefits and has been used to treat patients with a wide variety of ailments, including chronic pain, chemotherapy-induced nausea and vomiting, mental disorders (anxiety, insomnia, posttraumatic stress disorder, etc.), neurological disorders (epilepsy, amyotrophic lateral sclerosis, Huntington’s disease, Parkinson’s disease, multiple sclerosis, etc.), and many other medical conditions [5]. However, due to a lack of research and funding, there have been insufficient large-scale controlled trials to support these claims or use within the medical field [6,7,8,9,10,11,12].

Due to the rising usage of cannabis in the medical profession as well as increased legalization across the US, the public’s perceptions of how harmful marijuana use is has been changing. In fact, cannabis use is no longer considered risky behavior among most of America’s youth. However, there are real risks and adverse effects associated with cannabis use, especially in vulnerable populations such as adolescents and patients with psychotic disorders [6]. For example, cannabis use can cause permanent intelligent quotient (IQ) loss (as much as 8 points) when individuals begin consumption during adolescence [13,14]. Cannabis use can also negatively affect memory, problem-solving abilities, coordination, mood, and fetal growth, as well as has the potential to cause hallucinations, delusions, and psychosis [14,15,16,17]. In fact, studies have shown associations between cannabis use/CUD and an increased risk of psychiatric disorders and Reward Deficiency Syndrome (RDS) subsets such as schizophrenia, depression, anxiety, and substance use disorder [18]. Finally, cannabis use has been associated with worse educational outcomes, relationship problems, lower income, and reduced life satisfaction [14,19,20].

## 2. Neurogenetic and Epigenetic Aspects of Cannabinoids

**Figure 1:**
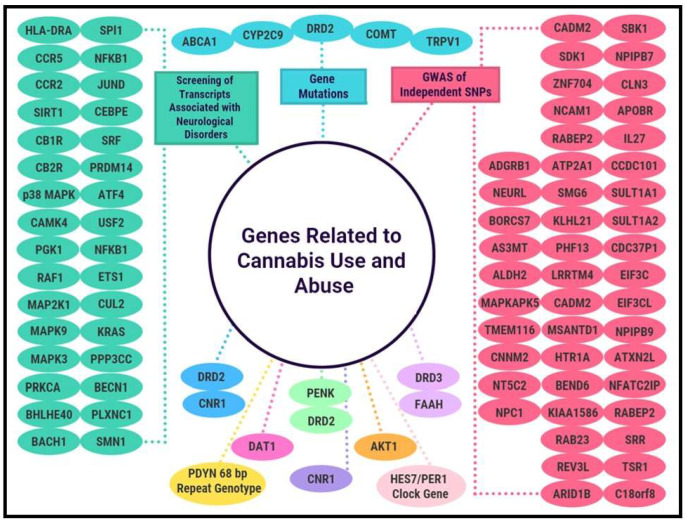
Genes Related to Cannabis Use and Abuse.

The emerging construct RDS is related to an umbrella terminology of all addictive, impulsive, and compulsive behaviors, including substances and non-substances, and was first coined by Blum’s group in 1995 [21]. It is a genetic and epigenetic phenomenon that leads to impairment of the brain reward circuitry, which results in hypo-dopaminergic function [22]. Genetics and epigenetics also appear to play a role in cannabis use and CUD (Table 1, Figure 1). In fact, research has shown that approximately 50–70% of liability to CUD and 40–48% of cannabis use initiation is the result of genetic factors [23,24]. One study, for example, found that cannabis use was both genetically and phenotypically correlated with self-harm and depression [25], both of which are subsets of Reward Deficiency Syndrome (RDS) [26,27]. In addition, another subset of RDS is schizophrenia [28]. The genetic liability for CUD appears strongly correlated with schizophrenia, above and beyond tobacco and cannabis ever-use, with mixed evidence supporting a causal relationship between schizophrenia and CUD [29].

Another well-known subset of RDS [30] is Attention-Deficit/Hyperactivity Disorder (ADHD), which is a severely impairing neurodevelopmental disorder with a global prevalence of 5% in children and adolescents and of 2.5% in adults [31]. ADHD is associated with a significantly increased risk of substance use, abuse, and dependence. ADHD has also been shown to have a strong genetic component, and the heritability of ADHD is approximately 70–80% [32]. Specifically, Artigas et al., based on a two-in-a-single variant association analysis (*rs145108385* and *rs4259397*), utilizing a two-sample Mendelian randomization approach, discovered evidence that ADHD is causal for lifetime cannabis use, with a 7.9 odds ratio for cannabis usage in people with ADHD compared to people without ADHD [32].

Addiction is a complicated and multi-factorial disease. Examining genetic variations at multiple loci and gene-gene interactions amongst them (epistasis) can reveal vital information about the causative factors of addiction. Isir et al., discussed the relationship between the 1359 G/A polymorphism of the Cannabinoid Receptor 1 (*CNR1*) gene and the Dopamine Receptor D2 (*DRD2*) gene polymorphisms, as well as the net effect of any potential epistasis on the cannabis addiction phenotype within the Turkish population [33]. The study concluded that in the cases of substance abuse, overlapping expressions of *CNR1* and *DRD2* are the causes of *CNR1-DRD2* interactions and that the various polymorphisms of *CNR1* and *DRD2* genes may play crucial roles in the nature of these interactions in terms of promoting or mitigating an individual’s cannabis addiction risk factor [34]. 

Support for a hypodopaminergic trait was similarly found in a study that examined the relationship between catechol-O-methyltransferase (*COMT*) polymorphism and premorbid cannabis use in Turkish male patients with schizophrenia. The study showed that the *Val/Val* genotype, which is associated with increased *COMT* activity, was found to be significantly higher in patients with premorbid cannabis use (88.9%) compared to those without (68.4%) [35]. Additionally, the *Val/Val* genotype group’s mean total positive and negative syndrome scale (PANSS) score was significantly higher than that of the patients with the Met allele. Therefore, when comparing the *Val* and *Met* genotypes, the homozygous *Val/Val* genotype is associated with the highest amount of *COMT* activity, followed by the *Val/Met* genotype, followed by the homozygous *Met/Met* genotype, which would be associated with the lowest amount of *COMT* activity. These results strongly suggest that the high activity of *Val158Met* (*COMT*) reduces the function of dopamine at the reward site of the brain, leading to hypodopaminergia and potential cannabis seeking behavior. Moreover, Batalla et al., observed that cannabis users exhibited alterations in hippocampal total and specific subregional volumes (i.e., cornu ammonis (CA) subfields 1–4) when compared to controls, as well as correlations between cannabis use levels and specific and total subregional volumes [36]. Furthermore, this study found that cannabis and Dopamine Transporter (*DAT1*) gene polymorphism (i.e., *9/9R* and *10/10R* alleles) affected the total hippocampal volume and the fissure subregion, indicating low and high levels of dopamine availability. These findings suggest that carriers with high dopamine transporter activity cause hypodopaminergia, increasing the likelihood of cannabis seeking behavior.

Gerra et al., found a significant correlation between cannabis use and the *rs1800497 Taq1A* of the Ankyrin Repeat and Kinase Domain Containing 1 (*ANKK1*) gene [37]. The *rs1800497 Taq1A* polymorphism of the *ANKK1* gene is considered to be one of the most widely researched polymorphisms regarding the genetics of behavior and addiction and has been linked with many substance use disorders [37,38]. Additionally, the *ANKK1* gene is closely linked to the *DRD2* gene [39]. In the study by Gerra et al., physical and emotional neglect were also found to have an impact on cannabis use, while parental care was found to be a protective factor. 

The *Taq1* gene has two alleles, *A1* and *A2*. The *A1* allele is located approximately 10 kb downstream of the *DRD2* gene and is linked to decreased striatal *DRD2* density and habitual alcohol use. Furthermore, individuals that are homozygous for this allele (i.e., *A1/A1*) are typically prone to hypodopaminergic states. One study that examined CUD in Nigeria showed how both *Taq1 A1/A1* and *A1/A2* genotypes impacted variance in Cannabis Use Disorder Identification Test (CUDIT) scores (10.2% and 5.1%, respectively) [40]. In this study, the distribution of the *A1* allele amongst the general population correlates with the previous reports in a southwestern Nigerian population. The result suggests that carrying just a single allele of the *A1* is enough to predict cannabis abuse, as shown by the allele association with CUDIT scores. In conclusion, Adedeji et al., discovered that carrying an *A1* allele is a significant predictor of CUD [40]. Additionally, while in some cases, heterosis seems to display the phenotype in question, for example, alcohol seeking [41], the authors concluded that double *A1* alleles appear to be a necessity for the prediction of dependence.

The idea of homozygosity as being an important culprit is further supported by the earlier work of Noble and Blum and associates [42]. They reported on subjects with *A2/A2*, *A1/A2*, and *A1/A1* alleles, and found a strong association between alleles and the density of *DRD2* binding sites. The number of binding sites of the *DRD2* receptors was significantly reduced in subjects with the *A1* allele, in which a high association with alcoholism was found. A progressively reduced number of binding sites was found in subjects with *A2/A2*, *A1/A2*, and *A1/A1* alleles. Individuals that were homozygous for *A1/A1* had the lowest number of binding sites, while individuals with *A2/A2* had the highest number. The polymorphic pattern of the *DRD2* gene and its differential expression of receptors suggests the involvement of the dopaminergic system in conferring susceptibility to at least one subtype of severe alcoholism.

In another investigation, a single nucleotide polymorphism in the gene encoding for fatty acid amide hydrolase (*FAAH*) *C385A* (*rs324420*) was analyzed. *FAAH* is an enzyme that hydrolyzes the endocannabinoid anandamide and associated amidated signaling lipids. In this study, *FAAH* was investigated to determine whether its variance was linked with changes in cravings and withdrawal after marijuana abstinence, changes in craving after cue exposure, or sensitivity to the acute effects of marijuana. Specifically, *C385A* variance was found to be significantly correlated with changes in withdrawal after abstinence as well as happiness after smoking marijuana, but not craving behavior [43]. 

Cannabis exposure during important developmental milestones has the potential to disrupt epigenetic programming and markers, resulting in long-lasting changes in gene function and intergenerational physiological consequences. Research has shown that the embryonic neural system patterning is vulnerable to maternal cannabis use [44,45,46,47,48,49,50]. Its consumption during pregnancy has also been linked to a higher risk of behavioral, cognitive, addiction vulnerability, and neuropsychiatric defects [17,44,51,52,53]. Research by Smith et al., showed that developmental and pre-gestational cannabis exposure changed epigenetic processes like DNA methylation and histone alterations, which have functional repercussions for gene expression. The fetal epigenetic programming of genes was suggestive of alterations in regions involved in the development of various neuropsychiatric disorders, such as autism, ADHD, schizophrenia, SUD, etc. Specifically, DiNieri et al., studied striatal dopamine in fetal brain specimens that had been exposed to cannabis in utero [54]. They found that through epigenetic mechanisms that are responsible for histone lysine methylation regulation, the expression of the messenger RNA for *DRD2* was reduced in the nucleus accumbens (NAc), which is a crucial reward center located in the ventral striatum. 

Important research by Hurd’s group revealed that prenatal and adolescent exposure to delta-9-tetrahydrocannabinol, the main psychoactive ingredient of cannabis, was associated with long-term effects on adult neurological systems relevant to psychiatric and substance use disorders [55]. Other epigenetic work by Oyaci et al., reported that when the methylation of the *DRD2* gene and the membrane-bound catechol-O-methyltransferase (*MB-COMT*) promoter in patients with CUD were compared with the control group, there was a significant difference between the *MB-COMT* promoter methylation status of the two groups [56]. Moreover, when *DRD2* gene methylation was compared to clinical features and *DRD2* genotype distribution in patients, the methylation status was found to be significantly different between the two groups due to the family history. Additionally, when the *MB-COMT* promotor methylation was compared to clinical features and *COMT Val158Met* genotype distribution in patients, the *MB-COMT* promoter methylation status was significantly different between the two groups due to the presence of alcohol use.

It is also important to recognize that adolescence represents a developmental period where there is a high risk of cannabis use experimentation. A study by Burgdorf et al., utilized a genetic knock-in mouse model (*FAAH^C/A^*) that biologically mimics the human polymorphism linked to problematic drug use [57]. This study showed that in adolescent female mice, *FAAH* polymorphism enhanced the mesolimbic dopamine circuitry projecting from the ventral tegmental area (VTA) to the NAc and altered *CB1* levels at excitatory and inhibitory terminals in the VTA. These cumulative developmental changes make adolescent female *FAAH^C/A^* mice more vulnerable to THC preference that lasts into adulthood. Overall, these findings suggest that this endocannabinoid genetic variant is a contributory factor to increased susceptibility to cannabis dependence in adolescent females.

**Table 1:** Summary of Relevant Neurogenetic Literature Related to Cannabis Use and Abuse.

Genes		Summary Findings	Reference
*COMT* *TRPV1* *CYP2C9* *DRD2* *ABCA1*		Findings in patients included mutations in genes *COMT* {odds ratio, 12 (95% confidence limit [CL], 1.3–88.1) *p* = 0.012}, transient receptor potential vanilloid receptor 1 (*TRPV1*) (odds ratio, 5.8 [95% CL, 1.2–28.4] *p* = 0.015), *CYP2C9* (odds ratio, 7.8 [95% CL, 1.1–70.1] *p* = 0.043), gene coding dopamine-2 receptor (*DRD2*) (odds ratio, 6.2 [95% CL, 1.1–34.7] *p* = 0.031), and ATP-binding cassette transporter gene (*ABCA1*) (odds ratio, 8.4 [95% CL, 1.5–48.1] *p* = 0.012).	[58]
*HLA-DRA* *CCR5* *CCR2* *SIRT1* *CB1R* *CB2R* *p38 MAPK* *CAMK4* *PGK1* *RAF1* *MAP2K1* *MAPK9* *MAPK3* *PRKCA* *BHLHE40* *BACH1*	*SPl1* *NFKB1* *JUND* *CEBPE* *SRF* *PRDM14* *ATF4* *USF2* *NFKB1* *ETS1* *CUL2* *KRAS* *PPP3CC* *BECN1* *PLXNC1* *SMN1*	The screening of a large number of transcripts associated with neurological disorders has shown that the effects of cannabis differed drastically between HIV− and HIV+ groups, particularly in gene networks playing a role in inflammation, neurodegeneration, apoptosis, and leukocyte adhesion and transmigration. The results indicate that cannabis, in the context of HIV, may have beneficial effects. However, in individual genes, the authors identified detrimental effects that were associated with polysubstance use as a covariate, particularly methamphetamine.	[59]
*CADM2* *SDK1* *ZNF704* *NCAM1* *RABEP2* *ATP2A1* *SMG6* *KLHL21* *PHF13* *LRRTM4* *CADM2* *MSANTD1* *HTR1A* *BEND6* *KIAA1586* *RAB23* *REV3L* *ARID1B* *ADGRB1* *NEURL* *BORCS7* *AS3MT* *ALDH2*	*SBK1* *NPIPB7* *CLN3* *APOBR* *IL27* *CCDC101* *SULT1A1* *SULT1A2* *CDC37P1* *EIF3C* *EIF3CL* *NPIPB9* *ATXN2L* *NFATC2IP* *RABEP2* *SRR* *TSR1* *C18orf8* *NPC1 TMEM116* *CNNM2* *NT5C2* *MAPKAPK5*	GWAS association results of independent SNPs that are significantly associated with lifetime cannabis use.	[60]
*DAT1*		These findings suggest that cannabis exposure alters the normal relationship between *DAT1* polymorphism and the anatomy of total and subregional hippocampal volumes and that specific hippocampal subregions may be particularly affected.	[36]
*HES7/PER1* Clock gene		*HES7/PER1* on chromosome 17 may represent a meaningful risk factor in the development of cannabis dependence and its severity.	[61]
*DRD2* *CNR1*		Results indicate that the increased phenotype of cases requires an individual to be either heterozygous at both loci or homozygous at locus B with homozygous risk factor *A1A1* present. We hypothesize that overlapping expressions of *CNR1* and *DRD2* are the causes of *CNR1-DRD2* interactions in cases of substance abuse, and the different polymorphisms of *CNR1* and *DRD2* genes may have decisive roles in the nature of these interactions in terms of promoting or alleviating the cannabis addiction risk factor of the individual.	[33,34]
*AKT1*		Genetic variation in *AKT1* may mediate both short-term as well as longer-term effects on psychosis expression associated with the use of cannabis, possibly through a mechanism of cannabinoid-regulated *AKT1/GSK-3* signaling downstream of the *DRD2* receptor.	[62]
*DRD2* *PENK*		The findings replicated the known association between the *rs6277 DRD2* SNP and decisions associated with negative reinforcement outcomes. Moreover, *PENK* variants (*rs2576573* and *rs2609997*) were significantly related to neuroticism and cannabis dependence.	[63]
*FAAH* *DRD3*		The association of reduced *FAAH* function with higher dopamine D3 receptors (*DRD3*) in human and mouse brains provides a mechanistic link between two brain systems that have been implicated in addiction-risk, especially cannabis.	[64]
*PDYN* 68 bp repeat genotype		This study provides the first data on how the *PDYN* 68 bp genotype is associated with gender-specific patterns of exposure to cannabis.	[65]
*CNR1*		The results are consistent with the role of cannabinoid receptors in the modulation of dopamine and cannabinoid reward pathways.	[66]

Table 1 provides a summary of some of the most relevant literature that demonstrates an association between various genes (associated polymorphisms) and cannabis use and dependence.

Figure 1 is a schematic representation of Table 1 and the various genes associated with cannabis use and abuse. 

## 3. Treatment of CUD

Currently, there is no established pharmacological treatment for CUD. The mainstay treatment for CUD relies on psychosocial interventions targeted at modifying behavior and offering support, such as cognitive behavioral therapy (CBT) or motivational interviewing [67,68,69]. Depending on the severity of the disorder and the patient’s needs, the aforementioned interventions can range from a one-time online visit and brief intervention in an outpatient setting to a more thorough treatment plan, including treatment of the disorder along with comorbidities in an outpatient or inpatient setting. Combination therapy (CBT + MET) is recommended if CBT or MET treatment is ineffective after a few weeks or the patient relapses after showing an initial response to treatment.

Currently, there is no FDA-approved medication for the treatment of CUD. However, several small-scale studies have shown that some medications may have a limited beneficial effect on reducing cannabis consumption. These medications include N-acetylcysteine [70], Gabapentin [71], Nabiximols [72,73], Cannabidiol [74], and Varenicline [75]. Although, there is inconsistent evidence of effectiveness among clinical trials regarding the use of N-acetylcysteine [76] and Nabiximols [77]. Finally, none of these drugs have been demonstrated to lead to prolonged abstinence or lessen the severity of CUD [78].

## 4. Use of Cannabis in Medical Therapies

There have been many debates, discussions, and published writings about the therapeutic value of the cannabis plant and the hundreds of cannabinoids it contains. Many states and countries have attempted, are attempting to, or have already passed bills to allow the legal use of cannabinoids, especially cannabidiol (CBD), as medicine to treat a wide range of clinical conditions without having been approved by a regulatory body. Therefore, by using PubMed and Google Scholar databases, we have reviewed published papers during the past 30 years on cannabinoids as medicines and commented on whether there is sufficient clinical evidence from well-designed clinical studies and trials to support the use of CBD or any other cannabinoids as medicines. Current research shows that CBD and other cannabinoids are currently not ready for formal indications as medicines in the treatment of a wide range of clinical conditions as promoted, with the exception of the limited use of CBD in the treatment of chemotherapy-induced or HIV/AIDS-associated nausea and vomiting, treatment-resistant epilepsy associated with three rare pediatric disorders, and CBD in combination with THC for treating multiple-sclerosis-associated spasticity [79,80,81,82,83,84,85,86,87,88,89]. 

Furthermore, evidence from the US also suggests that physicians frequently prescribe medical cannabis to patients who have conditions for which cannabis is contraindicated, such as CUD. In a US survey study, family doctors reported that 31% of their patients who were prescribed medical cannabis by another doctor had a medical condition that could be worsened by cannabis [90]. Another large-scale epidemiologic study found that, out of a total of 3784 respondents with past-year cannabis use, 32% of medical cannabis users had past-year CUD, compared with 25% of recreational cannabis users [91]. A US study of at-risk youth in Denver and San Francisco found that CUD was significantly (χ^2^ = 22.8, *p* < 0.001) associated with having a medical cannabis card [92].

Certainly, patients who use cannabis regularly should be assessed for CUD and given advice on avoiding cannabis-related harm. In addition, evidence-based practice standards and guidelines need to be established in order to prevent the overprescribing of cannabis [93]. Finally, research indicates that CBD and several other cannabinoids have the potential to treat multiple clinical conditions, but more pre-clinical, clinical studies, and clinical trials, which follow regulatory guidelines, are needed to formally recommend CBD and other cannabinoids as medicines [79,80,81,82,83,84,85,86,87,88,89].

## 5. Barriers to Cannabis Research

The US has been experiencing major shifts in public opinion and policy regarding the use of cannabis [94]. At the state level, there has been a significant increase in the legalization of medicinal and recreational cannabis. However, despite the increase in legalization, research into the potential therapeutic use and benefits of cannabis remains limited. This limitation is due to federal regulations, limited drug sources, and lack of funding, which impede cannabis research [95,96,97]. 

First, at the federal level, cannabis use is still illegal. Currently, cannabis is a Schedule 1 drug under the Controlled Substances Act. Schedule 1 drugs are defined as substances that have a high potential for abuse with no currently accepted medical use. Therefore, researchers must obtain FDA Investigational New Drug authorization and Drug Enforcement Administration (DEA) Schedule I registration, which can be a long and tedious process.

In terms of drug sources, cannabis for research purposes in the US is only available through the National Institute on Drug Abuse (NIDA) Drug Supply Program. Furthermore, for over 50 years, researchers were only allowed to use cannabis that was grown by a single facility at the University of Mississippi, which had a contract with NIDA. In 2021, the DEA finally decided to take steps to end the federal cannabis research monopoly, and now several independent companies have received conditional approvals for their applications to become federally authorized marijuana manufacturers for research purposes. This will allow for an increase in the supply of cannabis for research.

Funding for cannabis research is limited and many researchers claim it is biased. Recent data examining cannabis research funding found that the US spent 1.48 billion USD on cannabis research between 2000 and 2018 [98,99]. During this time, NIDA dominated cannabis research funding, allocating more than 1 billion USD to the field [98,99]. NIDA’s focus as an agency historically has been on drug abuse and addiction. Therefore, it should not be surprising that the agency has invested significantly more money into the research of cannabis misuse and its adverse effects than on the therapeutic benefits and potential of cannabis over the years. However, NIDA has begun to invest more into the therapeutic potential of cannabinoids and recently announced funding for a national registry of medical cannabis use and health outcomes [100]. While this is a step in the right direction, funding for the use of cannabis as a treatment option is still lacking. In fact, research examining the use of cannabinoids as a treatment option received more than 15 times the funding when compared to examining the use of cannabis as a treatment option [101].

With the need for researchers to obtain approval from three federal agencies (FDA, DEA, and NIDA), along with limited drug supply and funding, it can be challenging for them to acquire the type, quantity, and quality of cannabis needed for various types of cannabis research. Although NIDA has been implementing changes to help increase cannabis research, especially with regard to the potential therapeutic effects, the effects that these changes will have on cannabis research remain to be seen. Regardless, a broad network of sponsors is necessary to promote and support cannabis research that examines the harmful and therapeutic effects of cannabis and cannabinoids.

## 6. Conclusions

In summary, cannabis is one of the most widely used drugs in the world, and as legalization expands, so will the prevalence of cannabis use and, consequently, CUD. Research has shown that genetics and epigenetics play a significant role in cannabis use and CUD. Furthermore, the literature has shown significant links between cannabis use/CUD and an increased risk of psychiatric disorders and RDS subsets like schizophrenia, depression, anxiety, and substance use disorder. Comprehension of the genetic and epigenetic aspects of cannabinoids is required for future research, treatment plans, and the synthesis of pure compounds, which will be essential for FDA approval. In conclusion, having a better understanding of the genetic underpinnings of cannabis use and CUD will aid in the development of effective FDA-approved treatment therapies and the advancement of personalized medicine.
